# Fast and exact search for the partition with minimal information loss

**DOI:** 10.1371/journal.pone.0201126

**Published:** 2018-09-11

**Authors:** Shohei Hidaka, Masafumi Oizumi

**Affiliations:** 1 Japan Advanced Institute of Science and Technology, Nomi-shi, Ishikawa, Japan; 2 Araya Inc., Minato-ku, Tokyo, Japan; 3 RIKEN Brain Science Institute, Wako-shi, Saitama, Japan; Southwest University, CHINA

## Abstract

In analysis of multi-component complex systems, such as neural systems, identifying groups of units that share similar functionality will aid understanding of the underlying structures of the system. To find such a grouping, it is useful to evaluate to what extent the units of the system are separable. Separability or inseparability can be evaluated by quantifying how much information would be lost if the system were partitioned into subsystems, and the interactions between the subsystems were hypothetically removed. A system of two independent subsystems are completely separable without any loss of information while a system of strongly interacted subsystems cannot be separated without a large loss of information. Among all the possible partitions of a system, the partition that minimizes the loss of information, called the Minimum Information Partition (MIP), can be considered as the optimal partition for characterizing the underlying structures of the system. Although the MIP would reveal novel characteristics of the neural system, an exhaustive search for the MIP is numerically intractable due to the combinatorial explosion of possible partitions. Here, we propose a computationally efficient search to precisely identify the MIP among all possible partitions by exploiting the *submodularity* of the measure of information loss, when the measure of information loss is submodular. Submodularity is a mathematical property of set functions which is analogous to convexity in continuous functions. Mutual information is one such submodular information loss function, and is a natural choice for measuring the degree of statistical dependence between paired sets of random variables. By using mutual information as a loss function, we show that the search for MIP can be performed in a practical order of computational time for a reasonably large system (*N* = 100 ∼ 1000). We also demonstrate that MIP search allows for the detection of underlying global structures in a network of nonlinear oscillators.

## 1 Introduction

The brain can be envisaged as a multi-component dynamical system, in which each of individual components interact with each other. One of the goals of system neuroscience is to identify a group of neural units (neurons, brain area, and so on) that share similar functionality [1–4].

Approaches to identify such functional groups can be classified as “external” or “internal”. In the external approach, responses to external stimuli are measured under the assumption that a group of neurons share similar functionality if their responses are similar. A vast majority of studies in neuroscience have indeed used the external approach, by associating the neural function with an external input to identify groups of neurons or brain areas with similar functionality [5].

On the other hand, the internal approach measures internal interactions between neural units under the assumption that neurons with similar functionality are connected with each other. The attempts to measure internal interactions have rapidly grown following recent advancements in simultaneous recording techniques [6–8]. It is undoubtedly important to elucidate how neurons or brain areas interact with each other in order to understand various brain computations.

IIT states that the prerequisite for consciousness is integration of information realized by the internal interactions of neurons in the brain. IIT proposes to quantify the degree of information integration by an information theoretic measure, “integrated information” and hypothesizes that integrated information should be related to the level of consciousness.

**Fig 1:**
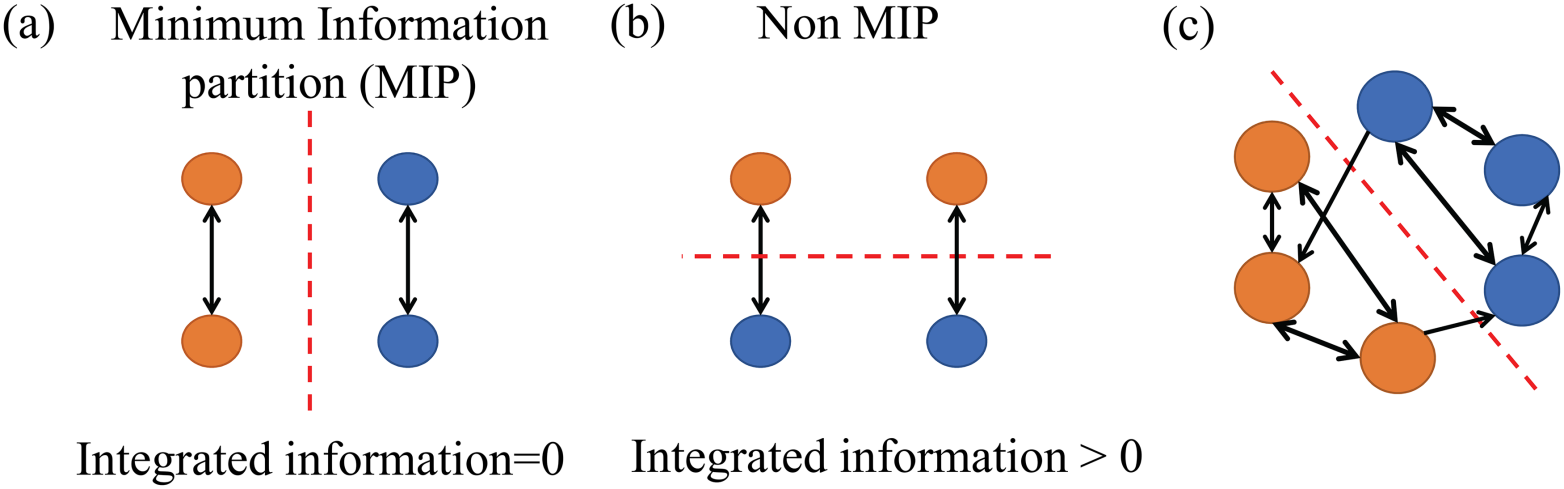
(a) Minimum information partition (MIP). (b) Another possible partition, which differs from the MIP. (c) MIP in a general network where there is no clear-cut partition.

In this study, we consider the problem of finding functional groups of neural units using the criterion of “minimal information loss”. Here, “information loss” refers to the amount of information loss caused by splitting a system into parts, which can be quantified by the mutual information between groups. For example, consider the system consisting of 4 neurons shown in Fig 1(a). The two neurons on each of the left and right sides are connected with each other but do not connect with those on the opposite side. The natural inclination is to partition the system into left (orange) and right (blue) subsystems, as shown in Fig 1(a). This critical partition can be identified by searching for the partition where information loss is minimal, i.e., mutual information between the two parts is minimal. In fact, if a system is partitioned with MIP as in the example system, information loss (mutual information between the subsystems) is 0 because there are no connections between the left (orange) and the right (blue) subsystems. If the system is partitioned in a different way than MIP, as shown in Fig 1(b), information loss is non-zero because there are connections between the top (orange) and the bottom (blue) subsystems. This is not the optimal grouping of the system from the viewpoint of information loss.

The concept of the partition with minimal information loss originated from Integrated Information Theory (IIT) [9–11] and the partition with minimal information loss is called “Minimum Information Partition (MIP)”. In IIT, information loss is quantified by integrated information [9–11], which is different from the mutual information we use in this study. Although the measure of information loss is different, we use the same technical term “MIP” in this study as well because the underlying concept is the same.

Although the theoretical idea of MIP is attractive to the fields of neuroscience as well as to network science in general, it has been difficult to apply it to the analysis of large systems. In a general case in which there is no obvious clear-cut partition (Fig 1(c)), an exhaustive search for the MIP would take an exceptionally large computational time which increases exponentially with the number of units. This computational difficulty has hindered the use of MIP-based characterization of a system.

In this study, we show that the computational cost of searching for the MIP can be reduced to the polynomial order of the system size by exploiting the submodularity of mutual information. We utilize one of the submodular optimization, the Queyranne’s algorithm [12], and show that the exponentially large computational time is drastically reduced to *O*(*N*^3^), where *N* is the number of units, when we only consider bi-partitions. We also extend the framework of the Queyranne’s algorithm to general *k*-partition and show that the computational cost is reduced to *O*(*N*^3(*k*−1)^). The algorithm proposed in this study is an exact search for the MIP, unlike previous studies which found only the approximate MIP [13, 14]. This algorithm makes it feasible to find MIP-based functional groups in real neural data such as multi-unit recordings, EEG, ECoG, etc., which typically consist of ∼100 channels.

In the review process of this paper, we found a previous study [15] that proposed the essentially same algorithm as the present study, i.e., Queyranne’s algorithm for bi-partition of mutual information. Compared with the previous study, this paper offers the following technical and conceptual advancement. As the technical advancement, we do not only propose the polynomial algorithm for bi-partition but also that for the exact minimal *k*-partition when *k* > 2, unlike an approximated algorithm proposed in the previous study. As the conceptual advancement, this is the first study to explicitly state the connection of Queyranne’s algorithm with the concept of MIP proposed in IIT and offer the possibility of utilizing Queyranne’s algorithm for the application of IIT.

The paper is organized as follows. In the Section 2, we formulate the search for the MIP, and show that mutual information is one of the submodular functions, and that we can treat it as a measure of information loss for a bi-partition. In Section 3, we report on numerical case studies which demonstrate the computational time of this MIP search for analysis of a system-wise correlation and also demonstrate its use for analysis of a nonlinear system. In Section 4, we discuss the potential use of the submodular search for other measures which are not exactly submodular.

## 2 Methods

### 2.1 Submodular function

For a ground set $X=\{x_{1},\ldots ,x_{N}\}$ of *N* elements and any pair of subsets $X,Y\in 2^{X}$, if a set function $f:2^{X}\mapsto R$ holds the inequality

$$\begin{array}{c} f(X)+f(Y)\geq f(X\cup Y)+f(X\cap Y), \end{array}$$ (1)

we call it *submodular* (See [16] for a review of submodularity). Equivalently, for *X* ⊆ *Y* ⊆ *Z* and *z* ∈ *Z* \ *Y*, a submodular set function *f* holds

$$\begin{array}{c} f(X\cup z)-f(X)\geq f(Y\cup z)-f(Y). \end{array}$$ (2)

If −*f*(*X*) is submodular, we call it *supermodular*.

Submodularity in discrete functions can be considered as an analogue of convexity in continuous functions. Intuitively, Eq (1) means that in some sense the sum of two components scores higher than the whole. Eq (2) means when something new is added to a smaller set, it has a larger increase in the function than adding it to a larger set. Also, the reader will be able to have the intuitive idea behind these inequalities by considering the special case, when the equality holds for *modular* function, that is both submodular and supermodular. For example, the cardinality of a set *f*(*X*) = |*X*| is modular, and holds equality for both Eqs (1) and (2).

It is easy to find the equivalence between the inequality Eqs (1) and (2): Apply Eq (1) to *X*′ = *X* ∪ *z* and *Y* such that *X* ⊆ *Y* ⊆ *Z* and *z* ∈ *Z* \ *Y*, and we have Eq (2). For converse, assume *X* ⊆ *Y* ⊆ *Z* and *z* ∈ *Z* \ *Y*, and apply Eq (2) to a series of paired sets, *X*_0_ := *X* and *Y*_0_ := *Y*, and *X*_*i*_ := *X*_*i*−1_ ∪ *z*_*i*_ and *Y*_*i*_ := *Y*_*i*−1_ ∪ *z*_*i*_ for every 0 < *i* ≤ |*Z* \ *Y*| and *z*_1_, *z*_2_, … ∈ *Z* \ *Y*. Then, we have Eq (1) by summing up these series of inequalities.

It has been shown that the minimization of submodular functions can be solved in polynomial order of computational time, circumventing the combinatorial explosion. In this study, we utilize submodular optimization to find the partition with minimal information loss (Minimum Information Partition (MIP)).

### 2.2 Minimum Information Partition (MIP)

We analyze a system with N∈N distinct components. Assume that each of the *N* components is a random variable, and denote the random variable of the *i*^th^ components by *x*_*i*_ for 1 ≤ *i* ≤ *N*. Denote the set of indices N:={1,2,…,N} and the set of the *N* variables by *V* = {*x*_1_, …, *x*_*N*_}. For the sake of simplicity, we consider bipartition of the whole system *V* for the explanatory purpose. We will deal with a general *k*-partition in Section 2.5. *V* is divided into two parts *M* and $\bar{M}$ where *M* is a non-empty subset of the whole system *V*, *M* ⊂ *V* and $\bar{M}$ is the complement of *M*, i.e., $\bar{M}=V\backslash M$. Note that bipartition (*V* \ *M*, *M*) of a fixed set *V* is uniquely determined by specifying only one part, *M*, because the other part is determined as the complement of *M*. Minimum Information Partition (MIP), *M*_MIP_, is defined as the subset that minimizes the information loss caused by partition, indicated by a non-empty subset *M* ⊂ *V*,

$$\begin{array}{c} M_{\text{MIP}}\left(V\right):=\underset{M\subset V,M\neq \emptyset }{\text{arg}\;\text{min}}f\left(M\right), \end{array}$$ (3)

where *f*(*M*) is the information loss caused by a bipartition specified by the subset *M*. More precisely, “MIP” defined in Eq 3 should be called “Minimum Information Bipartition (MIB)” because only bi-partition is taken into consideration. However, as we will show in Section 2.5, the proposed method is not restricted only to a bi-partition and can be extended to a general *k*-partition. To simplify terminology, we only use the term “MIP” through out the paper even when only bi-partition is considered.

The number of possible bi-partitions for the system size *N* is 2^*N*−1^ − 1, which grows exponentially as a function of the system size *N*. Thus, for even a modestly large number *N* of variables (*N* ∼ 40), exhaustively searching all bi-partitions is computationally intractable.

### 2.3 Information loss function

In this study, we use the mutual information between the two parts *M* and $\bar{M}$ as an information loss function,

$$f(M):=I(M;\bar{M}),$$ (4)

$$\begin{array}{cc} & =H\left(M\right)+H(\bar{M})-H(M,\bar{M}), \end{array}$$ (5)

where *H*(*X*) is the Shannon entropy [17, 18] of a random variable *X*,

$$\begin{array}{c} H\left(X\right):=-\sum_{x\in X}P\left(x\right)\log P\left(x\right). \end{array}$$

As we will show in the next section, the mutual information is a submodular function. The mutual information (Eq 5) is expressed as the KL-divergence between *P*(*V*) and the partitioned probability distribution $Q\left(V\right)=P\left(M\right)P\left(\bar{M}\right)$ where the two parts *M* and $\bar{M}$ are forced to be independent,

$$\begin{array}{c} I\left(M;\bar{M}\right)=D_{KL}(P\left(X\right)||P\left(M\right)P(\bar{M})). \end{array}$$ (6)

The Kullback-Leibler divergence measures the difference between the probability distributions and can be interpreted as the information loss when *Q*(*V*) is used to approximate *P*(*V*) [19]. Thus, the mutual information between *M* and $\bar{M}$ (Eq 6) can be interpreted as information loss when the probability distribution *P*(*V*) is approximated with *Q*(*V*) under the assumption that *M* and $\bar{M}$ are independent [11].

### 2.4 Submodularity of the loss functions

We will show that the mutual information (Eq 5) is submodular. To do so, we use the submodularity of entropy. The entropy *H*(*X*) is submodular because for *X* ⊂ *Y* ⊂ *Z* and *z* ∈ *Z* \ *Y*,

$$\begin{array}{cc} H(X\cup \{z\})-H(Y\cup \{z\}) & =-H(Y\backslash X|X\cup \{z\})\\ & \geq -H(Y\backslash X|X)\\ & =-H(Y)+H(X), \end{array}$$

which satisfies the condition of submodularity (Eq 2).

By straightforward calculation, we can find that the following identity holds for the loss function $f\left(M\right)=I\left(M;\bar{M}\right)$.

$$\begin{array}{c} f(A\cup B)+f(A\cap B)-f(A)-f(B)=H(A\cup B)+H(A\cap B)-H(A)-H(B)\\ +H\left(\bar{A}\cup \bar{B}\right)+H\left(\bar{A}\cap \bar{B}\right)-H\left(\bar{A}\right)-H\left(\bar{B}\right). \end{array}$$ (7)

Thus, from the submodularity of the entropy, the following inequality holds,

$$\begin{array}{c} f(A\cup B)+f(A\cap B)-f(A)-f(B)\leq 0, \end{array}$$ (8)

which shows that $f\left(M\right)=I\left(M;\bar{M}\right)$ is submodular.

### 2.5 MIP search algorithm

A submodular system (*V*, *f*) is said to be *symmetric* if *f*(*M*) = *f*(*V* \ *M*) for any subset *M* ⊆ *V*. It is easy to see that the mutual information is a symmetric submodular function from Eq 5. When a submodular function is symmetric, the minimization of submodular function can be solved more efficiently. Applying Queyranne’s algorithm [12] we can precisely identify the bi-partition with the minimum information loss in computational time *O*(*N*^3^). See also S1 File for more detail of the Queyranne’s algorithm.

We can extend the Queyranne’s algorithm for bi-partition to the exact search for a general *k*-partition with minimal information loss although it is more computationally costly. In what follows, we specifically explain 3-partition case for simplicity, but the argument is applicable to any *k*-partition in a form of mathematical induction. See also S2 File for more detail of the extension of the bi-partition algorithm.

## 3 Numerical studies

To demonstrate this search for the bi-partition with the minimal loss of information, we report here several case studies with artificial datasets. Throughout these case studies, we assume that the data is distributed normally. Under this assumption, we obtain the simple closed form

$$\begin{array}{c} f\left(M\right)=I(M;\bar{M})=\log_{2}|\Sigma_{M}|+\log_{2}|\Sigma_{\bar{M}}|-\log_{2}\left|\Sigma_{X}\right|, \end{array}$$ (9)

where Σ_*X*_ is the covariance matrix of the data, $\Sigma_{M},\Sigma_{\bar{M}}$ is the covariance matrix of the variables in the subsets *M* and $\bar{M}$, and |Σ| denotes the determinant of the matrix Σ. The computation of |Σ_*X*_| can be omitted because |Σ_*X*_| is constant across every step in the search and has no effect on the minimization of $I(M;\bar{M})$.

Our choice of the normality assumption above comes from computational feasibility for entropy or mutual information of high-dimensional joint variables. It is important to note that, for high-dimensional joint variables, such as mutual information between 15 variables and 15 other variables, the histogram-based empirical estimate would face two kinds of difficulties. First, it is difficult to compute due to its large number of support space, and second, the estimate for the high dimensional variable cannot avoid significant bias due to sample size relatively small to its support space. Suppose that we estimate mutual information of 15 dimensional variables, for instance, by the histogram-based approach with 10 equal-spaced bins for each of its marginal variable. In this case, we need an exponentially large number of bins (i.e., 10^15^) for the joint variable of the 15. For this large number of bins, it is difficult to directly compute the frequency, and the typical sample size (supposed to be < 10^8^) is too small to cover this large combinatorial space of 10^15^. Thus, histogram-based estimate of mutual information is not of practical use for high-dimensional variables. Therefore, we employed the normal-based estimate (Eq (9)). In the case study reported below, we will revisit this point and evaluate whether the two independent estimates of mutual information, normal-based and histogram-based one, match with each other in the bivariate case.

### 3.1 Study 1: Computational time

In the first case study, we compare the practical computational time of the submodular search with that of the exhaustive search. We artificially generated a dataset consisting of 10,000 points normally distributed over *N* dimensional space for *N* = 2, 3, …, 400. Each dimension is treated as an element in the set. The exhaustive search is performed up to *N* = 16, but could not run in a reasonable time for the dataset with *N* = 17 or larger due to limitations of the computational resource. Up to *N* = 16, we confirmed that the submodular search found the correct MIPs indicated by the exhaustive search.

**Fig 2:**
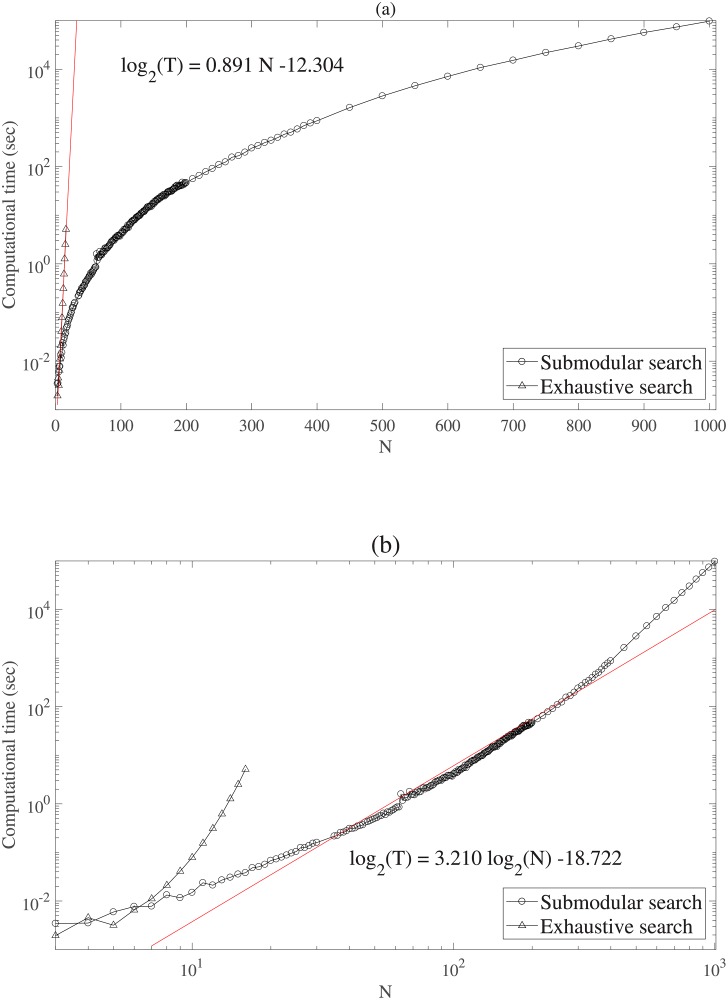
(a) The semi-logarithm plot and (b) log-log plot of the computation time for the two searches.

Fig 2(a) shows the semi-logarithm plot of the computation time of the two searches. The empirical computation time of the exhaustive search was closely along the line, log_2_ *T* = 0.891*N* − 12.304. This indicated that the exhaustive search took an exponentially large computational time ≈ *O*(2^*N*^), which fits with the number of possible bi-partitions. Fig 2(b) shows exactly the same results as the double-logarithm plot. In this plot, the computational time of the Queyranne’s search was closely along the line log_2_ *T* = 3.210 log_2_ *N* − 18.722, which indicated that the Queyranne’s search took cubic time ≈ *O*(*N*^3^), as expected from the theory. With *N* = 1000, the Queyranne’s search takes 9738 seconds of running time. The computational advantage of the Queyranne’s search over the exhaustive search is obviously substantial. For example, even with a modest number of elements, say *N* = 40, the computational time of the exhaustive search is estimated to be 1.07 × 10^7^ sec ≈ 123days while that of the Queyranne’s search is only 1sec.

### 3.2 Study 2: Toy example

**Fig 3:**
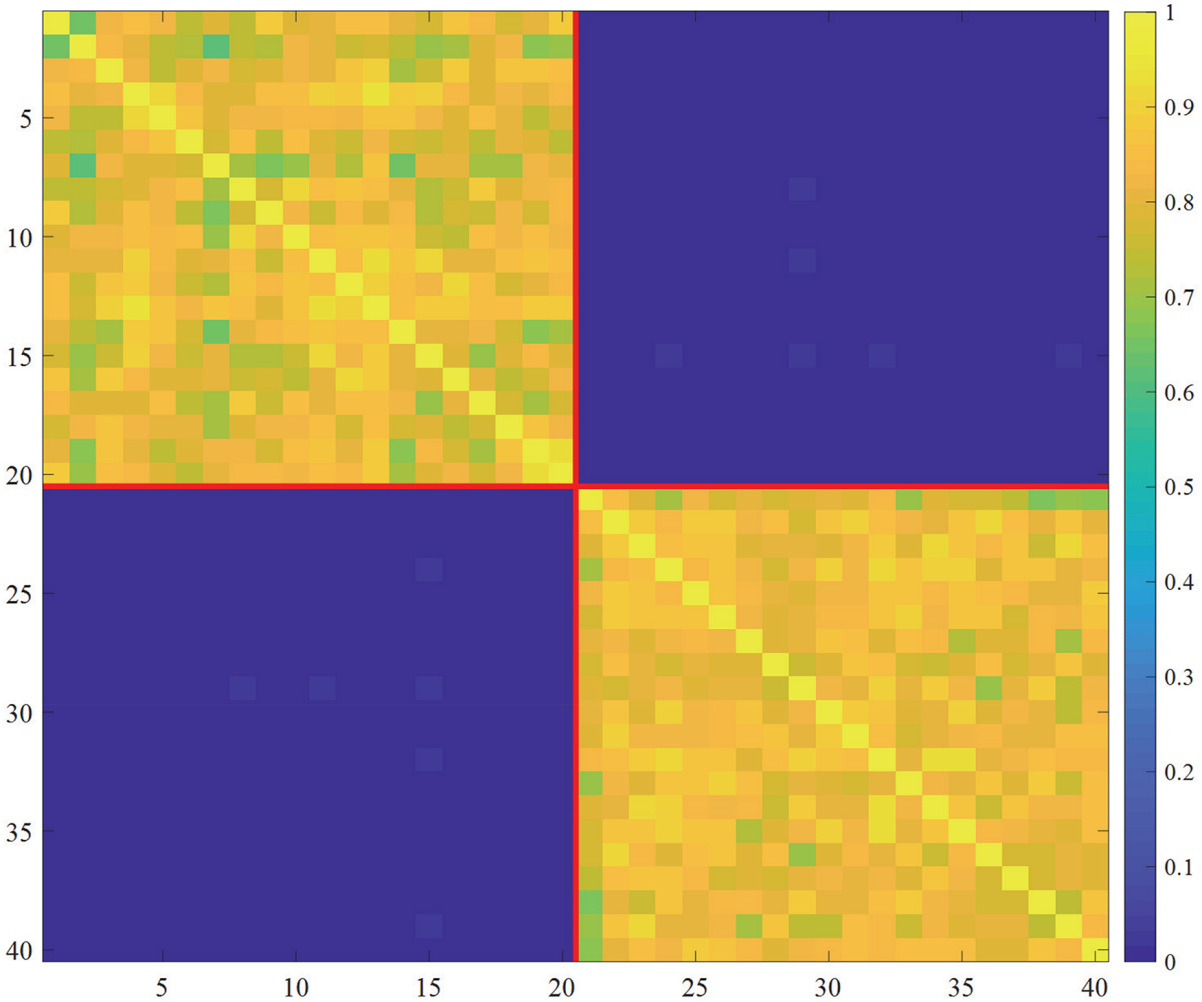
A correlation matrix which reflects two subsets of variables {1, 2, …, 20} and {21, 22, …, 40}.

As a demonstration of MIP search, we consider a set of 40 random variables with the correlation matrix shown in Fig 3. There are two subsets, variables 1, 2, …, 20, and 21, 22, …, 40, within each subset with positive correlations, while any other pairs of variables across the two subsets shows nearly zero correlation. This simulated dataset is supposed to capture the situation visualized in Fig 1(a) and 1(b). If the MIP search is successful, it would find the bipartition shown in Fig 1(a), in which each partitioned subset is either {1, 2} or {3, 4}.

The simulated correlation matrix is constructed as follows: We first generate two matrices $X_{1},X_{2}\in R^{1000\times 20}$ in which each of their elements is a normally distributed random value. For *i* = 1, 2, let $U_{i}S_{i}V_{i}^{T}=X_{i}$ be the singular value decomposition of the matrix *X*_*i*_, and construct another matrix *Y*_*i*_ := *U*_*i*_ *S*_*i*_(λ**1**_20,20_ + (1 − λ)*ϵ*_20,20_), where λ = 0.1, **1**_*k*,*k*_ is a *k* × *k* matrix with all element being 1, and *ϵ*_*k*,*k*_ is a *k* × *k* matrix with each element being a normally distributed random value. The forty dimensional dataset analyzed is constructed by concatenating the $Y=\left(Y_{1},Y_{2}\right)\in R^{1000\times 40}$, each of which is constructed in this way. By applying the MIP search, the system is partitioned into the pair of Variables 1, 2, …, 20, and the rest, as expected (the red line in Fig 3 indicates the found MIP for the dataset).

### 3.3 Study 3: Nonlinear dynamical systems

In Study 3, we demonstrate how MIP changes depending on underlying network structures. For this purpose, we chose a nonlinear dynamical system in which multiple nonlinear components are chained on a line. Specifically, we construct a series of variants of the Coupled Map Lattice (CML) [20]. Kaneko [20] analyzed the CML in which each component is a logistic map and interacts with the one or two other nearest components on a line, and showed the emergence of multiple types of dynamics in the CML. In this model, each component is treated as a nonlinear oscillator, and the degree of interaction between other oscillators can be manipulated parametrically. By manipulating the degree of interaction, we can continuously change the global structure of the CMLs from one coherent chain to two separable chains. We apply the MIP search for the CMLs with different interaction parameters, and test whether the MIP captures this underlying global structure of the network.

Specifically, the CML is defined as follows. Let us write the logistic map with a parameter *a* by *f*_*a*_(*x*) := 1 − *ax*^2^. Let *x*_*i*,*t*_ ∈ [0, 1] be a real number indicating the *i*^th^ variable at *t*^th^ time step for *i* = 1, 2, …, *N*, *t* = 0, 1, …, *T*. For each *i* = 1, …, *N*, the initial state of the variable *x*_*i*,0_ is set to a random number drawn from the uniform distribution on [0, 1]. For *t* > 0, we set the variables with the lateral connection parameter *ϵ* ∈ [0, 1] by

$$\begin{array}{ccc} x_{1,t} & = & \left(1-\epsilon \right)f_{a}\left(x_{i,t-1}\right)+\epsilon f_{a}\left(x_{i+1,t-1}\right),\\ x_{N,t} & = & \left(1-\epsilon \right)f_{a}\left(x_{i,t-1}\right)+\epsilon f_{a}\left(x_{i-1,t-1}\right),\\ x_{i,t} & = & \left(1-\epsilon \right)f_{a}\left(x_{i,t-1}\right)+\frac{\epsilon }{2}(f_{a}\left(x_{i-1,t-1}\right)+f_{a}\left(x_{i+1,t-1}\right))\;\text{for}\;1<i<N. \end{array}$$

**Fig 4:**
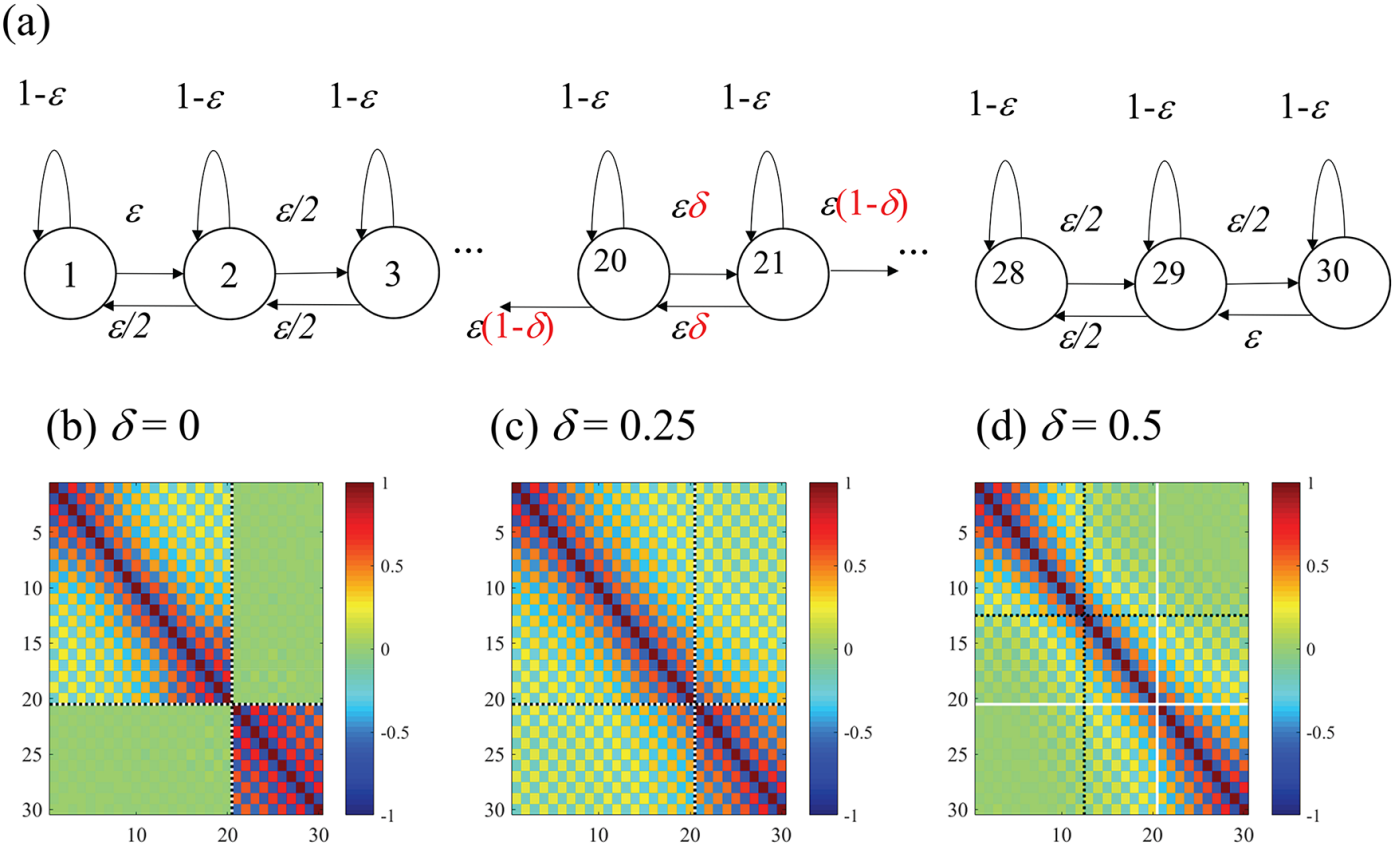
(a) The Coupled Map Lattice model with connection parameter *δ* indicating the connectivity between variables 20 and 21. The correlation matrices with (b) *δ* = 0 (disconnected), (c) *δ* = 0.25 (half-connected), and (d) *δ* = 0.5 (fully connected). The white crossing lines show the expected partition as the ground truth at which the parameter *δ* is manipulated, and the black dotted crossing lines show the MIP typically found for each particular parameter.

According to the previous study [20], the defect turbulence pattern in the spatio-temporal evolution in (*x*)_*i*,*t*_ is observed with the parameter *a* = 1.8950 and *ϵ* = 0.1. In this study, we additionally introduce the “connection” parameter between the variables *i* = 20, 21 among *N* = 30 variables (Fig 4(a)). Namely, with the connection parameter *δ*, we redefine variables 19, 20, 21 and 22 by

$$\begin{array}{ccc} x_{19,t} & := & \left(1-\epsilon \right)f_{a}\left(x_{19,t-1}\right)+\frac{\epsilon }{2}f_{a}\left(x_{18,t-1}\right)+\left(1-\delta \right)\epsilon f_{a}\left(x_{20,t-1}\right)\\ x_{20,t} & := & \left(1-\epsilon \right)f_{a}\left(x_{20,t-1}\right)+\frac{\epsilon }{2}f_{a}\left(x_{19,t-1}\right)+\delta \epsilon f_{a}\left(x_{21,t-1}\right)\\ x_{21,t} & := & \left(1-\epsilon \right)f_{a}\left(x_{21,t-1}\right)+\frac{\epsilon }{2}f_{a}\left(x_{22,t-1}\right)+\delta \epsilon f_{a}\left(x_{20,t-1}\right)\\ x_{22,t} & := & \left(1-\epsilon \right)f_{a}\left(x_{22,t-1}\right)+\frac{\epsilon }{2}f_{a}\left(x_{23,t-1}\right)+\left(1-\delta \right)\epsilon f_{a}\left(x_{21,t-1}\right). \end{array}$$

With the connection parameter *δ* = 1/2, this model is identical to the original CML above, and with *δ* = 0, it is equivalent to the two independent CMLs of (*x*_1_, …, *x*_20_) and (*x*_21_, …, *x*_30_), as it has no interaction between variable 20 and 21.

Given a sufficiently small connection parameter 0 ≤ *δ* < 1/2, we expect that the MIP would separate the system into the subsets {1, 2, …, 20} and {21, 22, …, 30}because the degree of the interaction between units 20 and 21 is the smallest. On the other hand, if the system is fully connected, which happens when *δ* = 1/2, we expect that the MIP would separate the system into the subsets {1, 2, …, 15} and {16, 17, …, 30} (in the middle of 30 units), due to the symmetry of connectivity on the line. The correlation matrices for different connection parameters *δ* from 0 to 1/2 are shown in Fig 4. In each matrix of Fig 4(b), 4(c) and 4(d), the crossed white lines show the expected separation between variables 20 and 21 at which the parameter *δ* is manipulated. We found the block-wise correlation patterns in the matrix with *δ* = 0, as expected (a typically found partition is depicted in the black dotted line in Fig 4(b), 4(c) and 4(d)); similar but less clear patterns with *δ* = 0.25; and no clear block-wise patterns with *δ* = 1/2.

**Fig 5:**
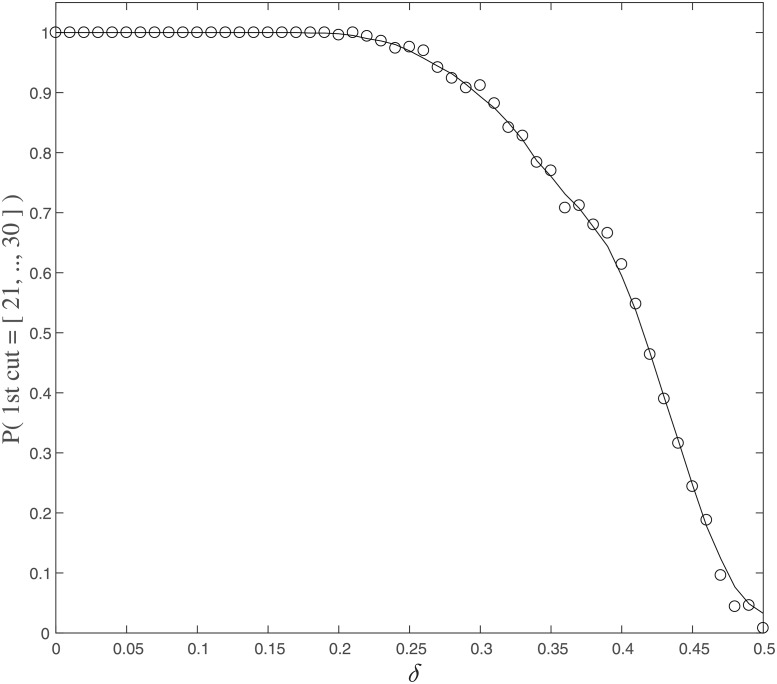
The probability of the subset with the smaller number of elements in the MIP is {21, 22, …, 30} is plotted as a function of the connection parameter *δ* ∈ [0, 1/2]. Each circle shows the sample probability of 500 independent simulations, and the solid line shows the moving average of the probability.

To summarize, this case study confirmed our theoretical expectation that the MIP captures the block-wise informational components; namely that the partition probability is a decreasing function of the connection parameter *δ* (Fig 5). This means that the MIP search detects the weakest underlying connection between 20 and 21, and successfully separates it into the two subsets, if the connections between 20 and 21 are weak.

For the technical validity, we add some remark on normality assumption in the computation of mutual information (Eq (9)). Most of the individual variables in the CML were found to be not exactly normally distributed, when the Kolmogorov-Smirnov test was performed on it. As we employ the normal-based estimate of mutual information as a practical approximation (for the difficulty of estimating mutual information in high-dimensional joint variables, see also the early discussion in Section 3), here we evaluate to what extent the normal-based estimate agree with the histogram-based one for pairs of variables, rather than its exact normality. For each pair of two distinct variables in the CML with the parameter *a* = 1.8950 and *δ* = 1/2, we computed empirical mutual information by drawing 10^7^ samples and construct histogram with the bins of 1000 equal-space intervals from the minimum to the maximum of the data. Then we compared the histogram-based estimate of mutual information with the normal-based one computed by Eq (9) treating the variables as the multivariate normal distribution. The correlation between the two types of estimates of mutual information computed from 435 pairs of variables was very high (0.9552). The high correlation coefficients between the two types of estimates suggest that the mutual information would be sufficiently approximated under the normality assumption although the whole shape of distribution is not a normal distribution.

## 4 Discussion

In this paper, we proposed a fast and exact algorithm which finds the weakest link of a network at which the network can be partitioned with the minimal information loss (MIP). Since searching for the MIP has the problem of combinatorial explosion, we employed Queyranne’s algorithm for a submodular function. We first showed that the mutual information is a symmetric submodular function. Then, we used it as an information loss function to which Queyranne’s algorithm can be applied. Our numerical case studies demonstrate the utility of the MIP search for a set of locally interacting nonlinear oscillators. This demonstration opens the general use of the MIP search for system neuroscience as well as other fields.

The proposed method can be utilized in Integrated Information Theory (IIT). In IIT, information loss is quantified by integrated information. To date, there are several variants of integrated information [9–11, 13, 21–24]. The mutual information was used as a measure of integrated information in the earliest version of IIT [9], but different measures which take account of dynamical aspects of a network were proposed in the later versions [10, 21]. To apply the proposed method, we first need to assess whether the other measures of integrated information are submodular or not. Even when the measures are not strictly submodular, the proposed algorithm may provide a good approximation of the MIP. An important future work is to assess the submodularity of the measures of integrated information, and also the goodness of the proposed algorithm as an approximation.

Another important issue in IIT is normalization of integrated information. The normalization factor was introduced in order to fairly compare different partitions that have different information sharing capacities across the partition [25]. In the earlier versions of IIT [9, 10], the MIP was defined as the partition where normalized integrated information is minimized. In this study, we did not consider normalization because the normalized mutual information is not submodular. For the application to IIT, it would be an interesting future work to assess whether the proposed algorithm works as a good approximation even when the measures of integrated information is normalized [26].

## Supporting information

S1 File Queyranne’s algorithm. (PDF)

S2 File Extension to *k*-partition algorithm. (PDF)
