# Supplementary material for: Fast and exact search for the partition with minimal information loss
Source: PLoS One. 2018 Sep 11;13(9):e0201126. doi: 10.1371/journal.pone.0201126 (PMC6133274; doi:10.1371/journal.pone.0201126)
Supplement: S1 File — (PDF) [file pone.0201126.s001.pdf]

Fast and exact search for the partition with minimal information loss

Shohei Hidaka[1*], Masafumi Oizumi[2,3],

1 Japan Advanced Institute of Science and Technology, Nomi-shi, Ishikawa, Japan
2 Araya Inc., Minato-ku, Tokyo, Japan
3 RIKEN Brain Science Institute, Wako-shi, Saitama, Japan

*shhidaka@jaist.ac.jp

Supporting information 1: Queyranne's algorithm

In this appendix, we briefly describe Queyranne's algorithm [1]. Suppose we have a submodular system (V, g) where V is a given set of elements and g is a submodular function defined for the power set of V. Let $f(U) := g(U) + g(V \setminus U)$ for every subset $U \subseteq V$ be a symmetric function constructed with the submodular function g. Queyranne's algorithm is used to search the subset U which minimizes the symmetric submodular function $f(U)$. For example, in this study, we consider the case that $f(U) = 2I(U; V \setminus U)$, identified up to a constant multiplier, and $g(U) = I(U; V \setminus U)$ are both mutual information (Eq. 2)

$$f(M) := I\left(M; \overline{M}\right), \tag{1}$$
$$= H(M) + H\left(\overline{M}\right) - H\left(M, \overline{M}\right). \tag{2}$$

In the algorithm proposed in [1], the key observation is that a special ordered pair (t, u), called a *pendent pair*, can be identified for an arbitrary subset $U \subseteq V$ in $O(N^2)$ time. Identification of a pendent pair (t, u) of the set V reduces the search space because for the desired subset U minimizing $f(U)$, either case (1) $U = \{u\}$ or (2) $U \supseteq \{t, u\}$ holds. Thus, by keeping case (1) as a candidate for the minimal partition, we can further refine case (2), in which we define a new ground set V' where the elements $\{t, u\}$ are treated as an inseparable unit element u'. By using the new merged element u', V' is defined as

$$V' := \{V \setminus \{t, u\}\} \cup \{u'\}.$$

After this procedure is applied once, the effective number of elements is reduced to $N - 1$. By applying this procedure recursively to search the set V' with $N - 1$ elements, we would obtain another candidate for the minimal partition and a candidate set V'' with $N - 2$ elements for further search. Thus, by finding the pendent pair for the given set V at each step recursively, we obtain $N - 1$ candidates for the minimal partition, and then find the minimal one from among them. In summary, this recursive computation takes $O(N^3)$ time because it requires the construction of a series of pendent pairs in $O(N^2)$, and $N - 1$ pendent pairs are needed to construct for minimization.

Next we illustrate the construction of a pendant pair. An ordered pair (t, u) of elements of V is called a pendent pair for (V, g), if $f(u)$ takes the minimum in all subsets of V which separate t from u, or equivalently

$$f(u) = \min\{f(U) \mid U \subset V, t \notin U \text{ and } u \in U\}.$$

There is at least one pendent pair for any symmetric submodular function. Further, a pendent pair can be constructed specifically for an element $x \in V$ as follows. For an element $x \in V$, let us write $v_1 := x$, $W_0 = \emptyset$, and $W_1 = \{v_1\}$. For $i > 1$,

$$v_i := \underset{u \in V \setminus W_{i-1}}{\arg\min}\ g(W_{i-1} \cup \{u\}) - g(\{u\}),$$

and $W_i := W_{i-1} \cup \{v_i\}$. For a set V of the size $N = |V|$, the (v_{N-1}, v_N) is a pendent pair. This construction of a pendent pair needs $O(N^2)$ times of evaluation of the function f. Importantly, for all $y \in V \setminus W_i$ and all $x \subseteq W_{i-1}$ in the series $(W_i)_{i=1}^N$ constructed by the procedure above for the submodular system (V, f), the following inequality holds

$$g(W_i) + g(y) \leq g(W_i \setminus X) + g(X + y).$$

See [1] for the proof of this inequality. By putting $i = N - 1$ in the inequality, we can see that the partition $(v_N, V \setminus \{v_N\})$ gives the minimum among all subsets separating v_N from v_{N-1}.

By definition of the pendent pair, one of the following two cases, case 1 or 2, holds for a given pendent pair (t, u).

1. The set $\{u\}$ is a solution of the minimization problem.

2. Some set $U \supseteq \{t, u\}$ is a solution of the minimization problem.

In the first case, the algorithm reports it. In the second case, the algorithm constructs another submodular system (V', f), in which a new element is defined by merging the pendent pair $u' = \{t, u\}$ and $V' = V \setminus \{t, u\} \cup u'$. The new system (V', f) with the merged pair is also submodular, and thus the same argument for the pendent pair can apply recursively.

References

1. Queyranne M. Minimizing symmetric submodular functions. Mathematical Programming. 1998;82(1-2):3–12.
