# Supplementary material for: Fast and exact search for the partition with minimal information loss
Source: PLoS One. 2018 Sep 11;13(9):e0201126. doi: 10.1371/journal.pone.0201126 (PMC6133274; doi:10.1371/journal.pone.0201126)
Supplement: S2 File — (PDF) [file pone.0201126.s002.pdf]

Fast and exact search for the partition with minimal information loss

Shohei Hidaka[1*], Masafumi Oizumi[2,3],

1 Japan Advanced Institute of Science and Technology, Nomi-shi, Ishikawa, Japan
2 Araya Inc., Minato-ku, Tokyo, Japan
3 RIKEN Brain Science Institute, Wako-shi, Saitama, Japan

*shhidaka@jaist.ac.jp

Supporting information 2: Extension to k-partition algorithm

The Queyranne's algorithm [1] works on minimization of $g(U) = f(U) + f(V \setminus U)$ with respect to non-empty set $U \subset V$ or $g((U, V \setminus U) = f(U) + f(V \setminus U)$ over bi-partition $(U, V \setminus U)$ with an arbitrary submodular set function f. Here we show a recursive method extending this symmetric submodular search over a set of bi-partitions to that of 3-partitions. The following argument will be easily extended to that of k-partition.

First let us denote the set of k-partitions of a given set V by

$$P_{k,V} := \left\{ (M_0, M_1, \ldots, M_{k-1}) | \bigcup_i M_i = V, M_i \cap M_j = \emptyset \text{ for any } i \neq j \text{ and, } M_i \neq \emptyset \text{ for every } i \right\}$$

For a submodular system (V, f) of a given underlying set V and a submodular set function $f : 2^V \mapsto \mathbb{R}$, we consider minimization of function $g : P_{3,V} \mapsto \mathbb{R}$ of the form

$$g\left((M_0, M_1, M_2)\right) = \sum_{i=0}^{2} f(M_i) + c, \tag{1}$$

where $c \in \mathbb{R}$ is a constant. This is an extension of the bi-partition function $g((U, V \setminus U) = f(U) + f(V \setminus U)$ to 3-partition function. In this section, we provide an algorithm to minimize this k-partition function by employing Queyranne's algorithm.

By defining $f(M) := H(M)$ for $M \subseteq V$, (V, f) is a submodular system, and the information loss function is written with a constant $c = -f(V)$ by

$$g\left((M_0, M_1, M_2)\right) = \sum_{i=0}^{2} f(M_i) - f(V).$$

For the special case $k = 2$, $g((M_0, M_1)) = I(M_0; M_1)$, this is identical to the minimal loss of information introduced in this study.

Our argument below does not depend on any specific form of a particular submodular function f, as long as the objective function takes the form in (1). The basic idea is to reduce the original objective function $g : P_{3,V} \mapsto \mathbb{R}$ to a set function $g_{3,V} : 2^V \mapsto \mathbb{R}$ by recursively defining $g_{2,U}$ for the remaining two subsets in a given bi-parition. As our goal is to minimize $g_{3,V}$, such reduction can be written specifically for non-empty $U \subset V$ by

$$g_{3,V}(U) := f_V(U) + h_{2,V}(U), \tag{2}$$

where for any $\emptyset \subset U_1 \subset U_2$, $f_{U_2}(U_1) := f(U_1) + f(U_2 \setminus U_1)$ and $h_{1,U_2}(U_1) := 0$ and

$$h_{2,U_2}(U_1) := \begin{cases} \min \begin{cases} \min_{\emptyset \subset U' \subset U_2 \setminus U_1} g_{2,U_2 \setminus U_1}(U') \\ \min_{\emptyset \subset U' \subset U_1} g_{2,U_1}(U') \end{cases} & (\min(|U_1|, |U_2 \setminus U_1|) > 1) \\ \min_{\emptyset \subset U' \subset U_2 \setminus U_1} g_{2,U_2 \setminus U_1}(U') & (|U_1| = 1) \\ \min_{\emptyset \subset U' \subset U_1} g_{2,U}(U') & (|U_2 \setminus U_1| = 1) \end{cases}. \quad (3)$$

This function (2) can be interpreted as recursive bi-partitioning across multiple stages: The first partition $(U, V \setminus U)$ of the set V is made on f_V, and the second partition $(U', \overline{U}')$ of either U or $V \setminus U$ on h_{k-1}, and so forth. For $|U| = 1$ or $|V \setminus U| = 1$, there is only one set for which the second partition can be made, otherwise smaller one of either $f_U(M_0)$ or $f_{V \setminus U}(M_0)$ has the solution. For $k = 2$, $g_{2,V}(U) = f_V(U)$, and minimization of $f_V(U)$ over the set of bi-partitions of V can be computed by the Queyranne's algorithm.

If this function $g_{3,V}$ is symmetric and submodular, we can apply the Queyranne's algorithm to this function at every recursive step above. Then, the minimum of $g_{3,V}(\hat{U})$ is identical to $g((\hat{U}, M_0, M_1))$ with the 3-partition is $(\hat{U}, \hat{M}_0, \hat{M}_1)$ such that

$$\hat{U} = \arg\min_{\emptyset \subset U \subset V} g_{3,V}(U) \text{ and } (\hat{M}_0, \hat{M}_1) = \arg\min_{(M_0, M_1) \in P_{2, V \setminus \hat{U}}} h_2(M_0, M_1)$$

or $(V \setminus \hat{U}, \hat{M}_0, \hat{M}_1)$ such that

$$\hat{U} = \arg\min_{\emptyset \subset U \subset V} g_{3,V}(U) \text{ and } (\hat{M}_0, \hat{M}_1) = \arg\min_{(M_0, M_1) \in P_{2, \hat{U}}} h_2(M_0, M_1).$$

As $g_{k,V}$ is obviously symmetric by definition, our main question now is whether it is submodular. The lemma following states that it is submodular.

Lemma 1. *For a given submodular system (V, f), the function $g_{3,V} : 2^V \mapsto \mathbb{R}$ is submodular.*

The proof of Lemma 1 needs Lemma 2 which is stated after the proof.

Proof. For a pair of set $X, Y \subseteq V$ and $k > 2$, consider the difference

$$\Delta := g_{3,V}(X) + g_{3,V}(Y) - g_{3,V}(X \cup Y) - g_{3,V}(X \cap Y),$$

and

$$\Delta_h := h_{3,V}(X) + h_{3,V}(Y) - h_{3,V}(X \cup Y) - h_{3,V}(X \cap Y).$$

As $\Delta - \Delta_h \geq 0$ due to submodularity of the function f, $\Delta_h \geq 0$ implies $\Delta \geq 0$ for any k. We show $\Delta_h \geq 0$ as follows. Now we will show that $\Delta_h \geq 0$, by using the fact that any function $g_{2,U}$ for $U \subseteq V$ is submodular.

Let us denote the minimizer of $g_{3,X}$ by

$$[X] := \arg\min_{\emptyset \subset W \subset X} g_{3,X}(W).$$

For all three cases of the function h (3), we consider the case below as the lower bound for Δ_h, and the other cases follow the essentially same argument:

$$\Delta_h \geq g_{3,X}([X]) + g_{3,Y}([Y]) - g_{3,X \cup Y}([X \cup Y]) - g_{3,X \cap Y}([X \cap Y])$$

By definition, $g_{3,X}([X]) \leq g_{3,X}(W)$ for any non-empty $W \subset X$, and thus for any non-empty $W \subset X \cup Y$ and $Z \subset X \cap Y$, we have

$$\Delta_{h,3} \geq g_{3,X}([X]) + g_{3,Y}([Y]) - g_{3,X \cup Y}(W) - g_{3,X \cap Y}(Z).$$

By Lemma 2, $\Delta_h \geq 0$ due to submodularity of $g_{2,U}$ for any $U \subseteq V$. □

The last line in the proof of Lemma 1 is shown as follows.

Lemma 2. *For an arbitrary submodular system* (V, f), $f(\emptyset) = 0$ *and* $\emptyset \subset W \subset Z \subseteq V$, *write*

$$f_Z(W) := f(W) + f(Z \setminus W).$$

For any given pair of partitions $(W, \overline{W}) \in P_{2,X\cup Y}$ *and* $(Z, \overline{Z}) \in P_{2,X\cap Y}$, *there is some pair* $(A, \overline{A}) \in P_{2,X}$ *and* $(B, \overline{B}) \in P_{2,Y}$ *which holds the inequality*

$$f_X(A) + f_Y(B) \geq f_{X\cup Y}(W) + f_{X\cap Y}(Z).$$

Vice versa, for any given pair of partitions $(A, \overline{A}) \in P_{2,X}$ *and* $(B, \overline{B}) \in P_{2,Y}$, *there is some pair* $(W, \overline{W}) \in P_{2,X\cup Y}$ *and* $(Z, \overline{Z}) \in P_{2,X\cap Y}$ *which holds the inequality above.*

This lemma states about a kind of submodularity on the bi-partition function f_X by existence of some pair W and Z for any pair A and B or vice versa. The proof of Lemma 2 is beyond the scope of this section, and will be reported elsewhere.

The Lemma 1 states that Queyranne's algorithm can be used to minimize the symmetric submodular set function $g_{m,U}(U')$ with respect to non-empty $U' \subset U$ at each step of the function $g_{k,V}(U)$. This observation further applies recursively for a k-partition problem, which can be reduced to the minimization of $(k-1)-$partition problem by defining an appropriate function. As minimization of a k-partition function includes the minimization of $(k-1)$-partition function, the computational time of this recursive algorithm for the minimal k-partition of n elements would take the order $O(n^{3(k-1)})$.

References

1. Queyranne M. Minimizing symmetric submodular functions. Mathematical Programming. 1998;82(1-2):3–12.
